# (*E*)-4-[(4-Diethyl­amino-2-hy­droxy­benzyl­idene)amino]­benzoic acid

**DOI:** 10.1107/S160053681200997X

**Published:** 2012-03-10

**Authors:** Hadi Kargar, Zahra Sharafi, Reza Kia, Muhammad Nawaz Tahir

**Affiliations:** a Department of Chemistry, Payame Noor University, PO Box 19395-3697 Tehran, I. R. of IRAN; b Department of Chemistry, Marvdasht Branch, Islamic Azad University, Marvdasht, Iran; c Department of Chemistry, Science and Research Branch, Islamic Azad University, Tehran, Iran; d Department of Physics, University of Sargodha, Punjab, Pakistan

## Abstract

In the title compound, C_18_H_20_N_2_O_3_, a potential bidentate *N*,*O*-donor Schiff base ligand, the benzene rings are inclined at an angle of 12.25 (19)°. The mol­ecule has an *E* conformation about the C=N bond. One of the ethyl groups is disordered over two positions, with a refined site-occupancy ratio of 0.55 (1):0.45 (1). An intra­molecular O—H⋯N hydrogen bond makes an *S*(6) ring motif. In the crystal, pairs of O—H⋯O hydrogen bonds link mol­ecules, forming inversion dimers with *R*_2_^2^(8) ring motifs.

## Related literature

For background to Schiff base ligands and their metal complexes, see: Kargar *et al.* (2011 ▶, 2012 ▶); Kia *et al.* (2010 ▶). For standard bond lengths, see: Allen *et al.* (1987 ▶). For hydrogen-bond motifs, see: Bernstein *et al.* (1995 ▶).
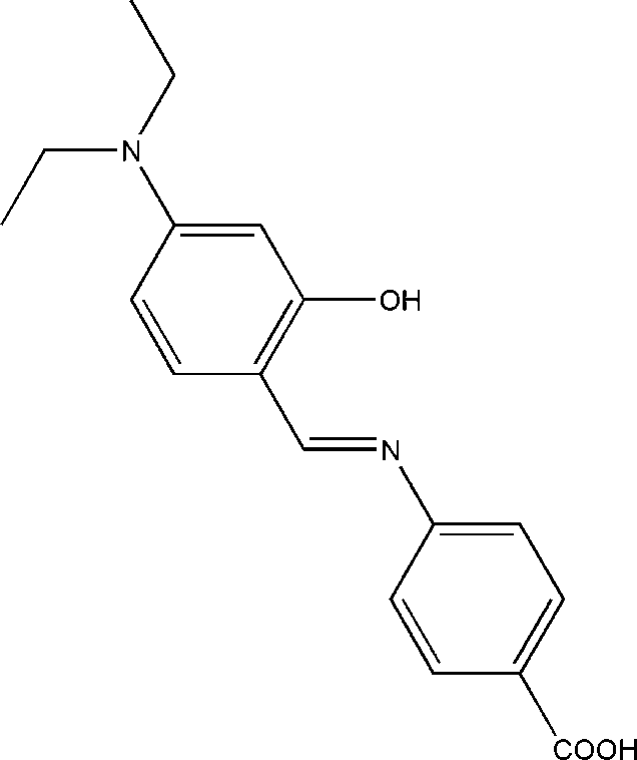


## Experimental

### 

#### Crystal data

C_18_H_20_N_2_O_3_
*M* *_r_* = 312.36
Monoclinic, 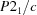

*a* = 12.4216 (8) Å
*b* = 8.1511 (6) Å
*c* = 16.0820 (11) Å
β = 93.001 (3)°
*V* = 1626.06 (19) Å^3^
*Z* = 4
Mo *K*α radiation
μ = 0.09 mm^−1^
*T* = 296 K
0.25 × 0.12 × 0.10 mm

#### Data collection

Bruker SMART APEXII CCD area-detector diffractometer
Absorption correction: multi-scan (*SADABS*; Bruker, 2005 ▶) *T*_min_ = 0.978, *T*_max_ = 0.991
11192 measured reflections
2872 independent reflections
918 reflections with *I* > 2σ(*I*)
*R*_int_ = 0.097

#### Refinement

*R*[*F* ^2^ > 2σ(*F* ^2^)] = 0.058
*wR*(*F* ^2^) = 0.133
*S* = 0.94
2872 reflections
219 parameters
H-atom parameters constrained
Δρ_max_ = 0.14 e Å^−3^
Δρ_min_ = −0.13 e Å^−3^

### 

Data collection: *APEX2* (Bruker, 2005 ▶); cell refinement: *SAINT* (Bruker, 2005 ▶); data reduction: *SAINT*; program(s) used to solve structure: *SHELXS97* (Sheldrick, 2008 ▶); program(s) used to refine structure: *SHELXL97* (Sheldrick, 2008 ▶); molecular graphics: *SHELXTL* (Sheldrick, 2008 ▶); software used to prepare material for publication: *SHELXTL* and *PLATON* (Spek, 2009 ▶).

## Supplementary Material

Crystal structure: contains datablock(s) global, I. DOI: 10.1107/S160053681200997X/su2388sup1.cif

Structure factors: contains datablock(s) I. DOI: 10.1107/S160053681200997X/su2388Isup2.hkl

Supplementary material file. DOI: 10.1107/S160053681200997X/su2388Isup3.cml

Additional supplementary materials:  crystallographic information; 3D view; checkCIF report

## Figures and Tables

**Table 1:** Hydrogen-bond geometry (Å, °)

*D*—H⋯*A*	*D*—H	H⋯*A*	*D*⋯*A*	*D*—H⋯*A*
O3—H3⋯N1	0.82	1.88	2.609 (3)	147
O2—H2⋯O1^i^	0.82	1.80	2.613 (2)	170

Symmetry code: (i) 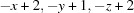.

**Fig. 1.**
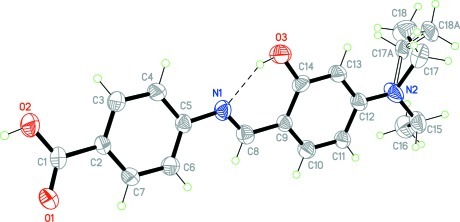
The ORTEP plot of the title compound, showing 40% probability displacement ellipsoids and the atomic numbering. The open bonds show the minor component of the disordered ethyl group. The dashed lines show the O-H···N hydrogen bond (see Table 1 for details).

**Fig. 2.**
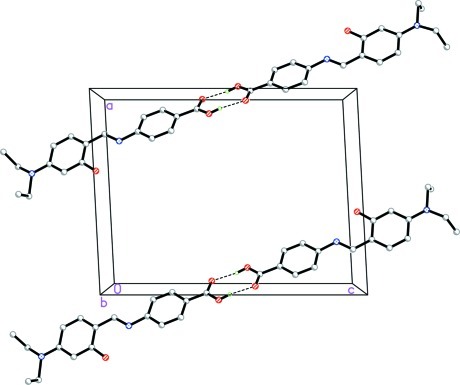
The crystal packing diagram of the title compound viewed along the b-axis, showing the inversion dimers with an R22(8) ring motif. Only the hydrogen atoms involved the hydrogen bonding are shown - see Table 1 for details.

## supplementary crystallographic information

### Comment

In continuation of our work on the crystal structure analysis of Schiff base ligands (Kargar *et al.*, 2011, 2012; Kia *et al.*, 2010), we determined the crystal structure of the title compound.

The asymmetric unit of the title compound, Fig. 1, comprises a potential bidentate N,O-donor Schiff base ligand. The bond lengths (Allen *et al.*, 1987) and angles are within the normal ranges. The intramolecular O3—H3···N1 hydrogen bond (Table 1) makes an S(6) ring motif (Bernstein *et al.*, 1995). The dihedral angle between the benzene rings is 12.25 (19)°, and the molecule has an E conformation about the C8═N1 bond. One of the ethyl groups was disordered over two position with a refined site occupancy ratio of 0.55 (1)/0.45 (1).

In the crystal, pairs of O—H···O hydrogen bonds (Table 1) link molecules into inversion dimers with an *R^2^_2_(8)* ring motif (Fig. 2).

### Experimental

The title compound was synthesized by adding 4-diethylaminosalicylaldehyde (2 mmol) to a solution of 4-carboxyaniline (2 mmol) in ethanol (30 ml). The mixture was refluxed with stirring for 30 min. The resultant solution was filtered. Pale-yellow single crystals of the title compound, suitable for *X*-ray structure analysis, were obtained by recrystallization from ethanol, by slow evaporation of the solvents at room temperature over several days.

### Refinement

The O-bound hydrogen atoms were located in a difference Fourier map and constrained to ride on the parent atoms with U_iso_(H) = 1.5 U_eq_(O). The rest of the hydrogen atoms were included in calculated positions and treated as riding atoms: C—H = 0.93, 0.96 and 0.97 Å for CH, CH_3_ and CH_2_ H atoms, respectively, with U_iso_ (H) = k × U_eq_(C), where k = 1.5 for CH_3_ H atoms, and = 1.2 for other H atoms. A rotating group model was applied to the methyl group. One of the ethyl groups was disordered over two position with a refined site occupancy ratio of 0.55 (1)/0.45 (1). Since the crystal was very small and not optimal and did not diffract significantly, the ratio of observed to unique reflections is only 32%.

### Crystal data

**Table:**

C_18_H_20_N_2_O_3_	*F*(000) = 664
*M**_r_* = 312.36	*D*_x_ = 1.276 Mg m^−^^3^
Monoclinic, *P*2_1_/*c*	Mo *K*α radiation, λ = 0.71073 Å
Hall symbol: -P 2ybc	Cell parameters from 3270 reflections
*a* = 12.4216 (8) Å	θ = 2.8–28.8°
*b* = 8.1511 (6) Å	µ = 0.09 mm^−^^1^
*c* = 16.0820 (11) Å	*T* = 296 K
β = 93.001 (3)°	Block, pale-yellow
*V* = 1626.06 (19) Å^3^	0.25 × 0.12 × 0.10 mm
*Z* = 4	

### Data collection

**Table:**

Bruker SMART APEXII CCD area-detector diffractometer	2872 independent reflections
Radiation source: fine-focus sealed tube	918 reflections with *I* > 2σ(*I*)
Graphite monochromator	*R*_int_ = 0.097
φ and ω scans	θ_max_ = 25.0°, θ_min_ = 2.5°
Absorption correction: multi-scan (*SADABS*; Bruker, 2005)	*h* = −14→12
*T*_min_ = 0.978, *T*_max_ = 0.991	*k* = −9→9
11192 measured reflections	*l* = −19→19

### Refinement

**Table:**

Refinement on *F*^2^	Primary atom site location: structure-invariant direct methods
Least-squares matrix: full	Secondary atom site location: difference Fourier map
*R*[*F*^2^ > 2σ(*F*^2^)] = 0.058	Hydrogen site location: inferred from neighbouring sites
*wR*(*F*^2^) = 0.133	H-atom parameters constrained
*S* = 0.94	*w* = 1/[σ^2^(*F*_o_^2^) + (0.0393*P*)^2^] where *P* = (*F*_o_^2^ + 2*F*_c_^2^)/3
2872 reflections	(Δ/σ)_max_ < 0.001
219 parameters	Δρ_max_ = 0.14 e Å^−^^3^
0 restraints	Δρ_min_ = −0.13 e Å^−^^3^

### Special details

**Table:**

Geometry. All esds (except the esd in the dihedral angle between two l.s. planes) are estimated using the full covariance matrix. The cell esds are taken into account individually in the estimation of esds in distances, angles and torsion angles; correlations between esds in cell parameters are only used when they are defined by crystal symmetry. An approximate (isotropic) treatment of cell esds is used for estimating esds involving l.s. planes.
Refinement. Refinement of F^2^ against ALL reflections. The weighted R-factor wR and goodness of fit S are based on F^2^, conventional R-factors R are based on F, with F set to zero for negative F^2^. The threshold expression of F^2^ > 2sigma(F^2^) is used only for calculating R-factors(gt) etc. and is not relevant to the choice of reflections for refinement. R-factors based on F^2^ are statistically about twice as large as those based on F, and R- factors based on ALL data will be even larger.

### Fractional atomic coordinates and isotropic or equivalent isotropic displacement parameters (Å^2^)

**Table:**

	*x*	*y*	*z*	*U*_iso_*/*U*_eq_	Occ. (<1)
C1	0.9492 (3)	0.4842 (5)	0.8861 (2)	0.0584 (12)	
C2	0.9076 (3)	0.4702 (5)	0.7993 (2)	0.0508 (11)	
C3	0.8265 (3)	0.3620 (5)	0.7757 (2)	0.0684 (13)	
H18	0.7990	0.2917	0.8150	0.082*	
C4	0.7858 (3)	0.3574 (5)	0.6946 (2)	0.0703 (13)	
H17	0.7299	0.2852	0.6801	0.084*	
C5	0.8256 (3)	0.4564 (5)	0.6347 (2)	0.0541 (12)	
C6	0.9084 (3)	0.5642 (5)	0.6578 (2)	0.0657 (13)	
H10	0.9371	0.6327	0.6184	0.079*	
C7	0.9481 (3)	0.5692 (5)	0.7398 (2)	0.0633 (12)	
H11	1.0036	0.6416	0.7547	0.076*	
C8	0.8135 (3)	0.5152 (5)	0.4913 (2)	0.0627 (13)	
H7	0.8806	0.5655	0.4982	0.075*	
C9	0.7599 (3)	0.5179 (5)	0.4096 (2)	0.0507 (11)	
C10	0.8031 (3)	0.5970 (5)	0.3437 (2)	0.0644 (12)	
H5	0.8700	0.6476	0.3520	0.077*	
C11	0.7519 (3)	0.6043 (5)	0.2663 (2)	0.0627 (12)	
H4	0.7842	0.6589	0.2234	0.075*	
C12	0.6503 (4)	0.5293 (5)	0.2515 (2)	0.0655 (13)	
C13	0.6062 (3)	0.4454 (5)	0.3171 (2)	0.0696 (14)	
H15	0.5408	0.3909	0.3083	0.084*	
C14	0.6582 (4)	0.4422 (5)	0.3949 (2)	0.0614 (12)	
C15	0.6378 (3)	0.6430 (6)	0.1074 (2)	0.0862 (15)	
H2A	0.6707	0.7408	0.1319	0.103*	
H2B	0.5769	0.6770	0.0713	0.103*	
C16	0.7176 (4)	0.5578 (5)	0.0570 (3)	0.1024 (17)	
H1A	0.7796	0.5285	0.0920	0.154*	
H1B	0.7391	0.6293	0.0134	0.154*	
H1C	0.6856	0.4604	0.0329	0.154*	
C17	0.4751 (11)	0.5013 (14)	0.1607 (7)	0.089 (5)	0.549 (11)
H17A	0.4373	0.5097	0.2118	0.107*	0.549 (11)
H17B	0.4407	0.5708	0.1182	0.107*	0.549 (11)
C18	0.4858 (16)	0.3342 (18)	0.1334 (11)	0.111 (6)	0.549 (11)
H18A	0.4156	0.2891	0.1200	0.167*	0.549 (11)
H18B	0.5212	0.2706	0.1771	0.167*	0.549 (11)
H18C	0.5277	0.3313	0.0850	0.167*	0.549 (11)
C17A	0.5142 (16)	0.405 (2)	0.1518 (10)	0.058 (5)	0.451 (11)
H17C	0.5183	0.3745	0.0937	0.070*	0.451 (11)
H17D	0.5286	0.3077	0.1855	0.070*	0.451 (11)
C18A	0.40179 (14)	0.4703 (4)	0.16718 (11)	0.075 (4)	0.451 (11)
H18D	0.3490	0.3878	0.1523	0.112*	0.451 (11)
H18E	0.3880	0.5665	0.1339	0.112*	0.451 (11)
H18F	0.3977	0.4977	0.2250	0.112*	0.451 (11)
N1	0.77425 (12)	0.44793 (19)	0.55449 (9)	0.0639 (11)	
N2	0.59881 (15)	0.5375 (3)	0.17449 (16)	0.0925 (14)	
O1	1.00875 (12)	0.59948 (19)	0.90912 (8)	0.0827 (10)	
O2	0.91877 (12)	0.37246 (19)	0.93606 (10)	0.0889 (10)	
H2	0.9476	0.3876	0.9825	0.133*	
O3	0.6110 (2)	0.3604 (4)	0.45575 (15)	0.0883 (10)	
H3	0.6443	0.3772	0.5003	0.132*	

### Atomic displacement parameters (Å^2^)

**Table:**

	*U*^11^	*U*^22^	*U*^33^	*U*^12^	*U*^13^	*U*^23^
C1	0.061 (3)	0.068 (3)	0.045 (3)	0.010 (3)	−0.004 (2)	0.005 (2)
C2	0.049 (3)	0.055 (3)	0.047 (3)	0.003 (2)	−0.007 (2)	−0.001 (2)
C3	0.078 (3)	0.078 (4)	0.049 (3)	−0.012 (3)	−0.002 (2)	0.009 (2)
C4	0.078 (3)	0.078 (4)	0.053 (3)	−0.026 (3)	−0.010 (3)	0.005 (3)
C5	0.065 (3)	0.053 (3)	0.044 (2)	0.003 (2)	−0.005 (2)	−0.004 (2)
C6	0.078 (4)	0.072 (3)	0.046 (3)	−0.016 (3)	−0.001 (2)	0.005 (2)
C7	0.068 (3)	0.066 (3)	0.055 (3)	−0.016 (2)	−0.009 (2)	0.000 (2)
C8	0.069 (3)	0.058 (3)	0.060 (3)	0.004 (2)	−0.007 (3)	−0.013 (2)
C9	0.056 (3)	0.051 (3)	0.044 (3)	0.000 (2)	−0.004 (2)	−0.003 (2)
C10	0.064 (3)	0.076 (3)	0.054 (3)	−0.005 (3)	0.004 (2)	0.003 (3)
C11	0.068 (3)	0.076 (3)	0.043 (3)	−0.010 (3)	−0.005 (2)	0.008 (2)
C12	0.064 (3)	0.081 (4)	0.050 (3)	−0.011 (3)	−0.008 (3)	0.006 (3)
C13	0.073 (3)	0.085 (4)	0.050 (3)	−0.024 (3)	−0.011 (3)	0.007 (3)
C14	0.077 (4)	0.058 (3)	0.049 (3)	−0.011 (3)	0.006 (3)	0.009 (2)
C15	0.076 (4)	0.121 (4)	0.060 (3)	0.000 (3)	−0.011 (3)	0.016 (3)
C16	0.111 (5)	0.116 (5)	0.079 (3)	0.000 (3)	−0.002 (3)	−0.005 (3)
C17	0.112 (15)	0.077 (10)	0.078 (7)	0.023 (9)	0.002 (7)	0.012 (7)
C18	0.138 (15)	0.088 (14)	0.109 (13)	−0.014 (10)	0.007 (9)	−0.011 (9)
C17A	0.076 (11)	0.055 (14)	0.040 (7)	0.008 (10)	−0.024 (7)	−0.009 (9)
C18A	0.034 (7)	0.100 (11)	0.090 (8)	0.013 (6)	0.000 (6)	0.013 (7)
N1	0.080 (3)	0.066 (3)	0.045 (2)	−0.0010 (19)	−0.007 (2)	0.0019 (19)
N2	0.071 (3)	0.155 (4)	0.050 (2)	−0.037 (3)	−0.015 (2)	0.022 (3)
O1	0.097 (2)	0.089 (2)	0.0590 (19)	−0.0284 (19)	−0.0243 (16)	0.0038 (17)
O2	0.124 (3)	0.090 (2)	0.0495 (18)	−0.023 (2)	−0.0197 (17)	0.0063 (17)
O3	0.105 (3)	0.105 (2)	0.0537 (18)	−0.042 (2)	−0.0093 (17)	0.0086 (19)

### Geometric parameters (Å, º)

**Table:**

C1—O1	1.240 (4)	C13—H15	0.9300
C1—O2	1.284 (4)	C14—O3	1.344 (4)
C1—C2	1.468 (4)	C15—N2	1.481 (4)
C2—C7	1.368 (4)	C15—C16	1.485 (5)
C2—C3	1.377 (4)	C15—H2A	0.9700
C3—C4	1.374 (4)	C15—H2B	0.9700
C3—H18	0.9300	C16—H1A	0.9600
C4—C5	1.369 (4)	C16—H1B	0.9600
C4—H17	0.9300	C16—H1C	0.9600
C5—C6	1.389 (4)	C17—C18	1.440 (19)
C5—N1	1.410 (4)	C17—N2	1.569 (14)
C6—C7	1.383 (5)	C17—H17A	0.9700
C6—H10	0.9300	C17—H17B	0.9700
C7—H11	0.9300	C18—H18A	0.9600
C8—N1	1.274 (4)	C18—H18B	0.9600
C8—C9	1.441 (4)	C18—H18C	0.9600
C8—H7	0.9300	C17A—C18A	1.53 (2)
C9—C10	1.374 (4)	C17A—N2	1.538 (15)
C9—C14	1.414 (5)	C17A—H17C	0.9700
C10—C11	1.370 (4)	C17A—H17D	0.9700
C10—H5	0.9300	C18A—H18D	0.9600
C11—C12	1.411 (5)	C18A—H18E	0.9600
C11—H4	0.9300	C18A—H18F	0.9600
C12—N2	1.365 (4)	O2—H2	0.8200
C12—C13	1.394 (5)	O3—H3	0.8200
C13—C14	1.379 (5)		
			
O1—C1—O2	122.8 (3)	O3—C14—C9	121.0 (4)
O1—C1—C2	121.2 (3)	C13—C14—C9	121.1 (4)
O2—C1—C2	116.0 (4)	N2—C15—C16	112.3 (4)
C7—C2—C3	118.4 (3)	N2—C15—H2A	109.1
C7—C2—C1	119.7 (4)	C16—C15—H2A	109.1
C3—C2—C1	121.9 (4)	N2—C15—H2B	109.1
C4—C3—C2	120.4 (4)	C16—C15—H2B	109.1
C4—C3—H18	119.8	H2A—C15—H2B	107.9
C2—C3—H18	119.8	C15—C16—H1A	109.5
C5—C4—C3	121.6 (4)	C15—C16—H1B	109.5
C5—C4—H17	119.2	H1A—C16—H1B	109.5
C3—C4—H17	119.2	C15—C16—H1C	109.5
C4—C5—C6	118.3 (3)	H1A—C16—H1C	109.5
C4—C5—N1	116.9 (4)	H1B—C16—H1C	109.5
C6—C5—N1	124.7 (4)	C18—C17—N2	96.6 (12)
C7—C6—C5	119.7 (4)	C18—C17—H17A	112.4
C7—C6—H10	120.1	N2—C17—H17A	112.4
C5—C6—H10	120.1	C18—C17—H17B	112.4
C2—C7—C6	121.6 (4)	N2—C17—H17B	112.4
C2—C7—H11	119.2	H17A—C17—H17B	110.0
C6—C7—H11	119.2	C18A—C17A—N2	109.6 (11)
N1—C8—C9	123.8 (4)	C18A—C17A—H17C	109.8
N1—C8—H7	118.1	N2—C17A—H17C	109.8
C9—C8—H7	118.1	C18A—C17A—H17D	109.8
C10—C9—C14	117.2 (4)	N2—C17A—H17D	109.8
C10—C9—C8	122.0 (4)	H17C—C17A—H17D	108.2
C14—C9—C8	120.8 (4)	C17A—C18A—H18D	109.5
C11—C10—C9	122.7 (4)	C17A—C18A—H18E	109.5
C11—C10—H5	118.7	H18D—C18A—H18E	109.5
C9—C10—H5	118.7	C17A—C18A—H18F	109.5
C10—C11—C12	120.2 (4)	H18D—C18A—H18F	109.5
C10—C11—H4	119.9	H18E—C18A—H18F	109.5
C12—C11—H4	119.9	C8—N1—C5	122.4 (3)
N2—C12—C13	121.8 (4)	C12—N2—C15	122.2 (3)
N2—C12—C11	120.3 (4)	C12—N2—C17A	117.5 (7)
C13—C12—C11	118.0 (4)	C15—N2—C17A	118.8 (7)
C14—C13—C12	120.8 (4)	C12—N2—C17	121.8 (5)
C14—C13—H15	119.6	C15—N2—C17	111.2 (5)
C12—C13—H15	119.6	C1—O2—H2	109.5
O3—C14—C13	117.9 (4)	C14—O3—H3	109.5
			
O1—C1—C2—C7	10.7 (6)	C12—C13—C14—C9	−2.7 (6)
O2—C1—C2—C7	−170.5 (3)	C10—C9—C14—O3	179.1 (4)
O1—C1—C2—C3	−167.8 (4)	C8—C9—C14—O3	−2.6 (6)
O2—C1—C2—C3	11.1 (6)	C10—C9—C14—C13	1.2 (6)
C7—C2—C3—C4	−1.5 (6)	C8—C9—C14—C13	179.5 (4)
C1—C2—C3—C4	176.9 (4)	C9—C8—N1—C5	−175.9 (3)
C2—C3—C4—C5	1.4 (6)	C4—C5—N1—C8	−169.4 (3)
C3—C4—C5—C6	−0.5 (6)	C6—C5—N1—C8	14.6 (5)
C3—C4—C5—N1	−176.7 (3)	C13—C12—N2—C15	172.2 (4)
C4—C5—C6—C7	−0.1 (6)	C11—C12—N2—C15	−9.0 (5)
N1—C5—C6—C7	175.8 (3)	C13—C12—N2—C17A	−21.8 (10)
C3—C2—C7—C6	0.9 (6)	C11—C12—N2—C17A	157.0 (9)
C1—C2—C7—C6	−177.6 (4)	C13—C12—N2—C17	18.6 (7)
C5—C6—C7—C2	−0.1 (6)	C11—C12—N2—C17	−162.5 (6)
N1—C8—C9—C10	177.6 (4)	C16—C15—N2—C12	88.2 (4)
N1—C8—C9—C14	−0.6 (6)	C16—C15—N2—C17A	−77.6 (10)
C14—C9—C10—C11	0.0 (6)	C16—C15—N2—C17	−115.7 (5)
C8—C9—C10—C11	−178.2 (4)	C18A—C17A—N2—C12	97.2 (10)
C9—C10—C11—C12	0.2 (6)	C18A—C17A—N2—C15	−96.3 (11)
C10—C11—C12—N2	179.6 (4)	C18A—C17A—N2—C17	−9.9 (8)
C10—C11—C12—C13	−1.5 (6)	C18—C17—N2—C12	−98.4 (11)
N2—C12—C13—C14	−178.3 (4)	C18—C17—N2—C15	105.4 (10)
C11—C12—C13—C14	2.8 (6)	C18—C17—N2—C17A	−4.7 (18)
C12—C13—C14—O3	179.4 (4)		

### Hydrogen-bond geometry (Å, º)

**Table:**

*D*—H···*A*	*D*—H	H···*A*	*D*···*A*	*D*—H···*A*
O3—H3···N1	0.82	1.88	2.609 (3)	147
O2—H2···O1^i^	0.82	1.80	2.613 (2)	170

Symmetry code: (i) −*x*+2, −*y*+1, −*z*+2.
